# Characterization of a novel dual murine model of chemotherapy-induced oral and intestinal mucositis

**DOI:** 10.1038/s41598-023-28486-3

**Published:** 2023-01-25

**Authors:** Ali I. Mohammed, Antonio Celentano, Rita Paolini, Jun T. Low, Michael J. McCullough, Lorraine A. O’ Reilly, Nicola Cirillo

**Affiliations:** 1 Melbourne Dental School, The University of Melbourne, 720 Swanston Street, Carlton, VIC 3053 Australia; 2 College of Dentistry, The University of Tikrit, Tikrit, Iraq; 3 The Walter and Eliza Hall Institute of Medical Research, 1G Royal Parade, Parkville, VIC 3052 Australia; 4 Department of Medical Biology, University of Melbourne, Parkville, VIC 3000 Australia

**Keywords:** Mucositis, Diarrhoea, Chemotherapy

## Abstract

Oral and intestinal mucositis are debilitating inflammatory diseases observed in cancer patients undergoing chemo-radiotherapy. These are devastating clinical conditions which often lead to treatment disruption affecting underlying malignancy management. Although alimentary tract mucositis involves the entire gastrointestinal tract, oral and intestinal mucositis are often studied independently utilizing distinct organ-specific pre-clinical models. This approach has however hindered the development of potentially effective whole-patient treatment strategies. We now characterize a murine model of alimentary tract mucositis using 5-Fluorouracil (5-FU). Mice were given 5-FU intravenously (50 mg/kg) or saline every 48 h for 2 weeks. Post initial injection, mice were monitored clinically for weight loss and diarrhea. The incidence and extent of oral mucositis was assessed macroscopically. Microscopical and histomorphometric analyses of the tongue and intestinal tissues were conducted at 3 interim time points during the experimental period. Repeated 5-FU treatment caused severe oral and intestinal atrophy, including morphological damage, accompanied by body weight loss and mild to moderate diarrhea in up to 77.8% of mice. Oral mucositis was clinically evident throughout the observation period in 88.98% of mice. Toluidine blue staining of the tongue revealed that the ulcer size peaked at day-14. In summary, we have developed a model reproducing the clinical and histologic features of both oral and intestinal mucositis, which may represent a useful in vivo pre-clinical model for the study of chemotherapy-induced alimentary tract mucositis and the development of preventative therapies.

## Introduction

Mucositis is a common and most debilitating complication associated with the cytotoxicity of radiotherapy and chemotherapy^1^. The condition affects the entire alimentary canal from the mouth to the anus, precipitating ulceration, and pain in both the oral cavity and intestinal tract. Ancillary associated complications include anorexia, vomiting, and diarrhea^2–4^ which remain a significant burden for 40–80%^5,6^ of patients undergoing chemotherapeutic or radiation regimens (CRT)^7–10^. Crucially, mucositis has been reported as the most undesirable and bothersome side effect experienced by patients undergoing multiple types of cancer therapy^11^. Dose-limiting toxicity may be a consequence in some cases, which can lead to a delay or even cessation of otherwise durable cancer treatment, negatively impacting the benefits of chemotherapy^7^.

Chemotherapy-induced mucositis (CIM) affects both the mouth (oral mucositis, OM) and lower gastrointestinal tract (intestinal mucositis, IM), and for this reason often referred to collectively as alimentary tract mucositis. Assessment of mucositis often focuses on OM, partly due to the practical difficulties in directly studying gastrointestinal tissues from cancer patients^7,8,12^. In addition, IM is primarily assessed by clinical symptoms or biomarkers since it cannot be visually inspected^13,14^. Oral ulcerative lesions can progress and are associated with severe pain requiring opioid usage. In addition, the consequences of OM are far-reaching and include changes to nutritional status, gastrostomy or parenteral feeding dependence, hospitalization, and the predisposition to bacteraemia or sepsis in severe cases. This sequel adversely affects patient’s quality of life and are compounded by the necessary recruitment of additional health resources^7,15–17^.

Although mucositis severity and duration depend on the type of treatment, drug therapy schedule, and patient-based variables^18–21^, the vast majority of patients receiving CRT will develop mucositis, making it both a predictable and potentially preventable condition. Surprisingly, despite its widespread and clinically devastating consequences, there is currently little to offer patients in the way of effective treatment to prevent or mitigate mucositis^22^. One of the reasons for this slow clinical translation could be the paucity of a comprehensive animal and pre-clinical model designed to concurrently assess both oral and intestinal mucosal injury. Thus, an appropriate disease model is essential for the development of effective preventive and/or mitigative treatment strategies.

Animal models of mucositis (including radiotherapy- and chemotherapy-induced) have been widely used recently as a suitable alternative to human preclinical studies, providing novel insights for understanding mucositis pathobiology, as well as assessing novel interventions^23,24^. Most of the existing CIM murine models use 5-Flurouracil (5-FU) injections and are based on either a single high dose of 5-FU (100–500 mg/kg) or a smaller daily dose (30–50 mg/kg) for a maximum of 4 weeks^25–27^. While, rodent 5-FU injection protocols that cause intestinal mucositis are easy to conduct, unfortunately, the 5-FU injection protocol is insufficient to induce oral mucositis in mice unless head and neck radiation^28^, local mechanical, or chemical injuries are additionally applied^29,30^. As such, research has predominantly focused on either oral or intestinal mucosal damage as single entities, using diverse experimental designs and clinical or histopathological assessments^31–36^. There is an urgent need for a more holistic experimental animal model that is suitable for the assessment of both oral and gastrointestinal damage induced by chemotherapy.

The clinical course of OM is generally predictable and influenced by the kinetics of the anticancer therapy^37^. Early clinical signs of chemotherapy-induced OM appear approximately 3 to 4 days after the drug infusion with ulceration appearing shortly thereafter. The severity of OM peaks around two weeks and generally resolves spontaneously by 21 days after the infusion. The frequency of OM progression, severity, and recovery time depends on the toxicity of the chemotherapy protocol^38,39^.

In the intestine, chemotherapy disrupts cell division in the crypts of Lieberkühn and renewal of the villus epithelium leading to a rapid loss of both intestinal structure and functionality^4,40^. IM is characterized by the shortening of villi and disruption of crypt cell homeostasis. Clinical symptoms including abdominal pain, nausea, vomiting, abdominal bloating, diarrhea, constipation, and subsequent weight loss result from mucositis emanating in the small and large intestine^41,42^.

The aim of this study was to establish an experimental animal model suitable for the concurrent assessment of both oral and gastrointestinal damage induced by chemotherapy. Our work documents the characterization of an innovative mouse model of intravenous chemotherapy-induced alimentary tract mucositis reproducing the clinical, histopathological and inflammatory responses of both oral and intestinal human mucosa to 5-FU. To our knowledge, this is the most thorough simultaneous characterization of the oral and intestinal mucosal response to 5-FU published to date in a murine model, including clinical monitoring of OM onset, diarrhea severity in animals as a surrogate marker of gastrointestinal mucositis, the oxidative stress markers, inflammatory responses, as well as assessment of the integrity of the mucosal barrier. Importantly, this is the first study to simultaneously assess the deleterious effects of this type of chemotherapy on neutrophils, which may be associated with increased susceptibility to oral opportunistic infections in cancer chemotherapy patients.

## Results

### 5-FU treatment induces body weight loss and reduced survival

**Figure 1:**
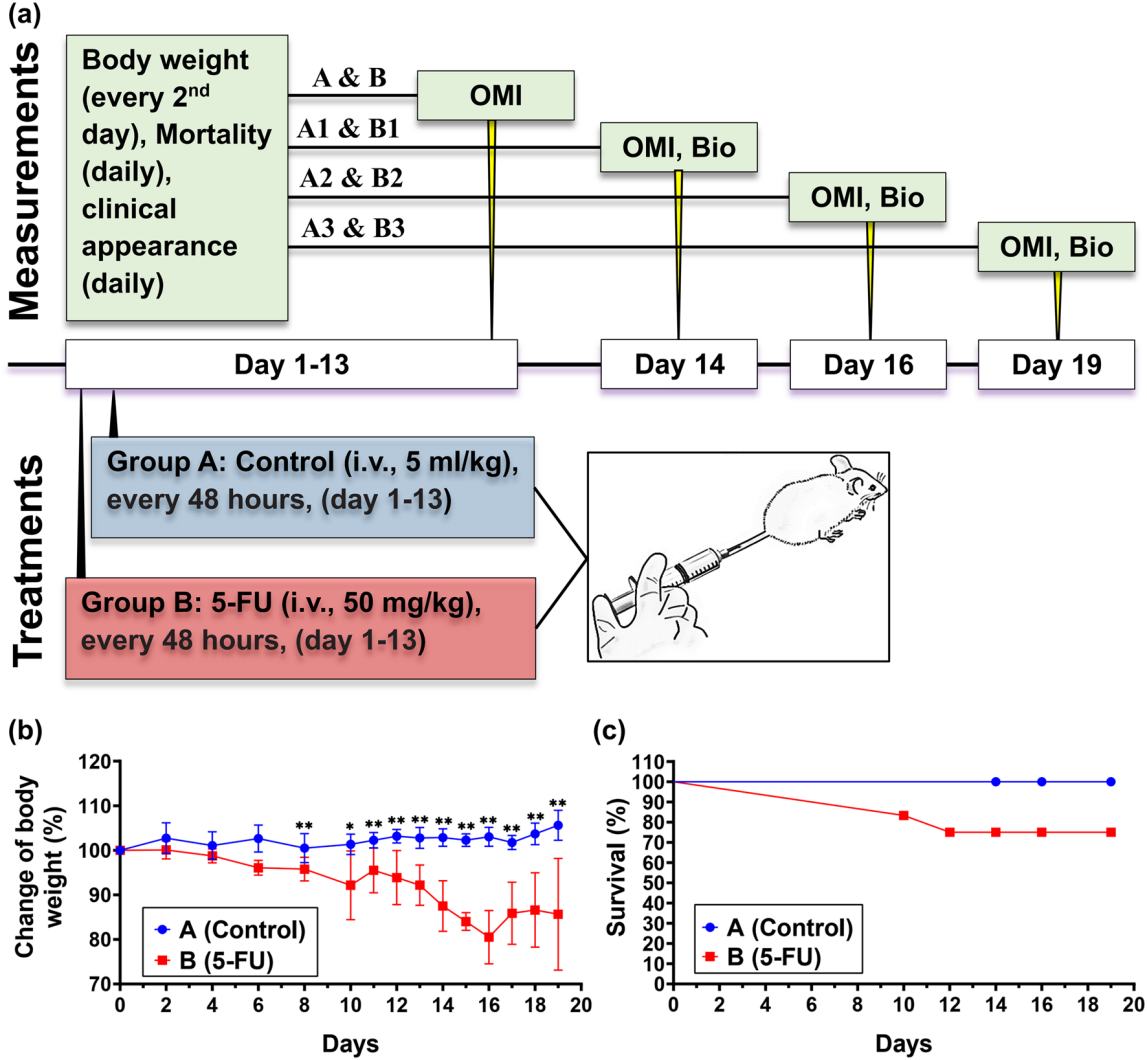
5-FU reduces body weight and survival of mice. (**a**) Schematic of the experimental procedure. 6–12 weeks-old C57BL/6 female mice were divided into 2 groups: Group A; Saline as normal control; Group B; 5-FU as positive control. 5-FU (50 mg/kg/day) was injected intravenously at 2-day intervals, starting from day 1 to day 13. Group A received physiological saline (the vehicle of 5-FU, 5 ml/kg/day). At days 14, 16, and 19 mice were sacrificed, and the tongue and the jejunum, were retrieved at necropsy. (**b**) Changes in body weight (BW) over time during 5-FU administration; BW of mice was measured every 24–48 h. The BW percentage change of each mouse was calculated and compared with the percent of BW at day 0. The mean BW of every group at day-0 was defined as 100%. Data are represented as mean ± standard error of mean (SEM). Multiple *t* tests were used to determine the statistical significance between the two groups. (**c**) Time-course of survival during 5-FU treatment. Logrank test was used for comparative analysis of survival rates. *P < 0.01 versus 5-FU group; **P < 0.001 versus 5-FU group; (*n* = 9 and 12 for group A and B, respectively).

In order to investigate the adverse effects associated with the administration of 5-FU, six-week-old C57BL/6 female mice were divided into two groups, A (control, *n* = 9) and B (5-FU, *n* = 12), according to the experimental plan summarized in Fig. 1a. The first measurable sign of mucositis is weight loss, which was recorded every 24–48 h. We observed maximal body BW loss in group B on day 16 after 5-FU induction (*p* < 0.001) (Fig. 1b). In addition, the BW (Fig. 1b) was significantly reduced in 5-FU-treated mice (group B) compared to those in the control group (A) at all timepoints measured from day 8 post 5-FU initiation. Weight loss in mice receiving 5-FU was also accompanied by a decrease in overall survival compared to control group (A), which showed no fatality (Fig. 1c). In accordance with our ethical approach, treatment was reduced to 50% levels in mice with weight loss (> 15%)and mice with a reduction of BW (> 20%) were immediately sacrificed (total *n* = 3; *n* = 2 mice, day 10, *n* = 1, day 12, included in the survival figures). Therefore, all subsequent analyses included 9 control mice and 9 5-FU-treated mice.

### Assessment of chemotherapy-induced gastrointestinal mucositis

**Figure 2:**
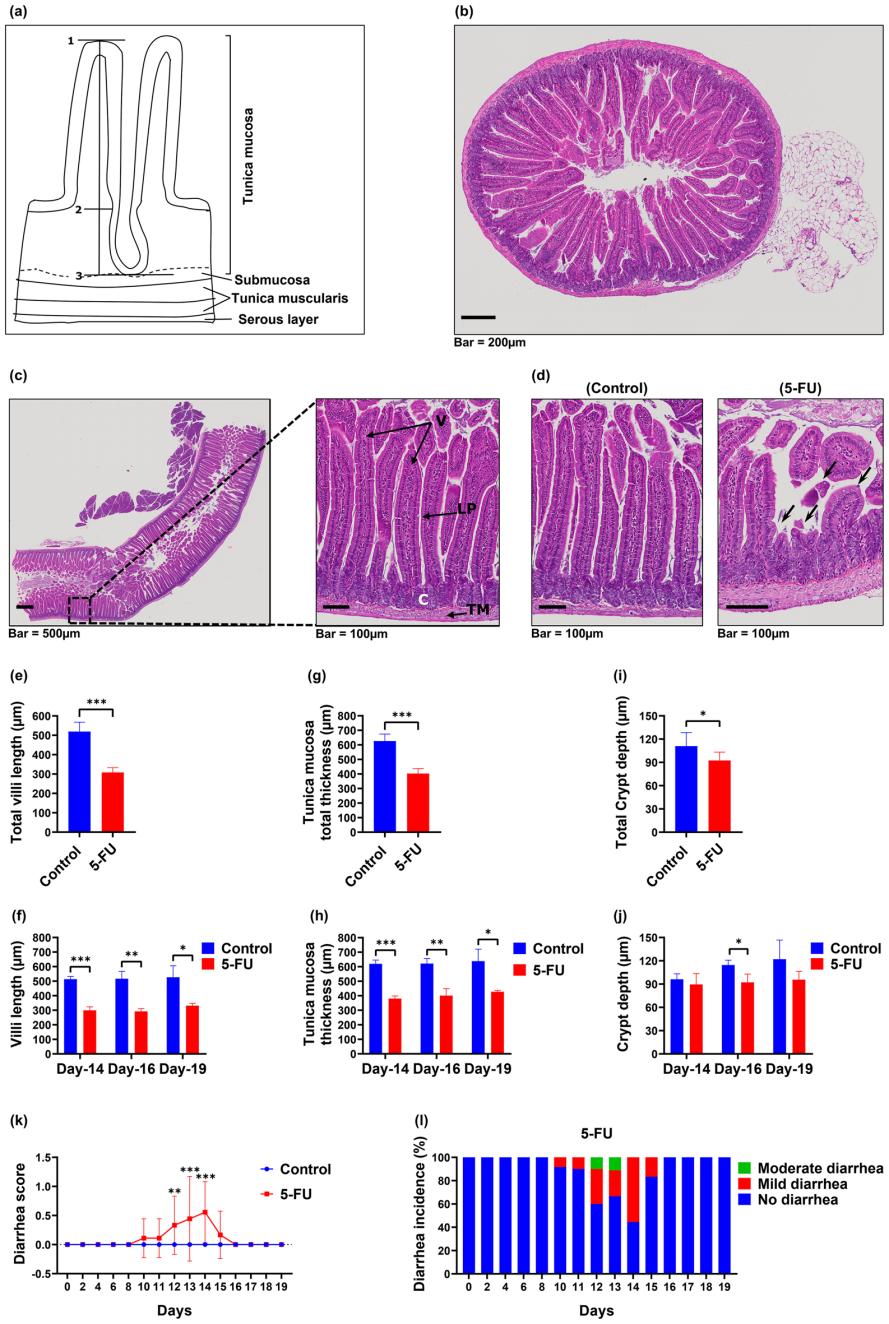
5-FU-induces intestinal mucosa damage in mice. Histological changes in the intestinal villi were determined using hematoxylin and eosin (H&E) staining. (**a**) Diagram of the wall of the small intestine demonstrating the morphometric measurements of the tunica mucosa, intestinal villi and crypts of the ileum. 1–3: Thickness of the tunica mucosa; 1–2: Length of the intestinal villi; 2–3: Depth of the intestinal crypt. (**b**) Representative microphotograph showing the histologic section of a transversely cut intestinal segment of the control animal. (**c**) Representative microphotograph showing the histologic section of a longitudinally cut intestinal segment of a control animal. Structural landmarks: Villi (V), Crypts (C), lamina propria (LP), Tunica muscularis (TM). (**d**) Representative microphotograph showing histological appearance of the intestinal mucosa of control, and 5-FU-treated mice. Compared with control mice, the innermost layer was largely destroyed, and the epithelial thickness reduced (epithelial atrophy), with villus length shortening and thinning of lamina propria (black arrows), accompanied by inflammatory cell infiltration. Identical histological aspects were confirmed in multiple control, and 5-FU-treated mice (*n* = 3, and 3, respectively). (**e**) Total villi length (average) of the mice jejunum between Day-14 and Day-19. (**f**) The average villi length of the jejunum at each time point. (**g**) Total thickness of tunica mucosa (average) of the mice jejunum between Day-14 and Day-19. (**h**) The average tunica mucosa thickness of the jejunum at each timepoint. (**i**) Total crypt depth (average) of the mice jejunum between Day-14 and Day-19. (**j**) The average crypt depth of the jejunum at each time point. Unpaired student *t* test was used to determine the statistical significance between groups. Data represented as mean ± SD. *p < 0.05. **p < 0.005. ***p < 0.001; *n* = 9 mice/group, 3 mice/group/timepoint, 12 representative measurement/tissue. (**k**) Diarrhea score. Score: 0, no diarrhea; 1, mild diarrhea; 2, moderate diarrhea; 3, severe diarrhea. Multiple *t* tests were used to determine the statistical significance between the two groups. The data shown are means ± SD. **P < 0.005 versus control group (A); *** *P* < 0.001 versus control group (A). (**l**) Incidence (%) and severity of diarrhea following 5-FU treatment. The occurrence of mild and moderate diarrhea was observed in mice treated with 5-FU.

Microscopical, histological and clinical parameters were used to ascertain the onset and the severity of gastrointestinal mucositis. The morphologic features of the murine intestine of experimental mice were assessed by morphometric analysis of formalin fixed paraffin embedded (FFPE) H&E stained jejunum sections (mid- gastrointestinal tract). For this analysis of the jejunum intestinal wall, tissue samples were oriented longitudinallyto assess tunica mucosa thickness, intestinal villi length and crypt depth (Fig. 2a).

Mice in the control group (A) showed no histopathological changes during the experimental time-course; the epithelium of the intestinal mucosa was intact with normal appearance of villi and intestinal crypts (Fig. 2b,c,d). In contrast, 5-FU treatment resulted in a marked reduction in the integrity and architecture of intestinal mucosa, demonstrating epithelial atrophy, villus length reduction and thinning of the lamina propria accompanied by inflammatory cell infiltration (Fig. 2d). In addition, total villus length was significantly decreased in the jejunum of the 5-FU group (B) (308.09 ± 24.72 µm) compared to control group (A). (519.28 ± 48.42 µm, *P* < 0.0001, Fig. 2e), with villus length reduction peaking at day 16 (Fig. 2f, Table 1). Morphometric analysis of H&E stained jejunum revealed a significant reduction in the total combined thickness of the intestinal tunica mucosa in the 5-FU group (B) compared to the control group (A) between days 14–19 (Fig. 2g,h). Since crypt depth modifications are further indicators of intestinal epithelial architecture damage, analysis of crypt depth revealed a significant reduction in total crypt depth observed in the 5-FU (B) group (days 14–19) compared to the control group (A) (Fig. 2i). In this case, the most significant change was observed on day 16 (*p* < 0.05, Fig. 2j, Table 1).

**Table 1:** 5-FU-induced intestinal injury. Descriptive statistics of morphometric variables of the jejunum wall of C57BL/6 mice following chemotherapy (5-FU) treatment. Groups were treated with saline (5 ml/kg/day) via tail vein (i.v.) (control group A), and 5-FU (50 mg/kg/day) i.v. (group B, 5-FU). Presented data are means ± SD of 10 representative measurement/tissue (three representative mice/group). a denoted *p* < 0.05 compared to control group (A).

Treatment groups	Morphometric measurement (µm)		
	Tunica mucosa thickness	Villi length	Crypts depth
Control			
Day-14	619.93 ± 25.48	513.61 ± 19.18	96.19 ± 6.76
Day-16	621.60 ± 43.52	517.07 ± 50.91	114.54 ± 6.14
Day-19	638.11 ± 82.98	527.16 ± 79.18	122.04 ± 24.59
Dya14-19	626.55 ± 47.53	519.27 ± 48.42	110.92 ± 17.47
5-FU			
Day-14	380.40 ± 17.94^a^	299.95 ± 23.73^a^	89.59 ± 14.09
Day-16	400.82 ± 48.24^a^	292.55 ± 18.78^a^	92.13 ± 10.72 ^a^
Day-19	427.67 ± 8.24^a^d	331.79 ± 14.98^a^	95.63 ± 10.83
Dya14-19	402.97 ± 33.18^a^	308.09 ± 24.72^a^	92.45 ± 10.70 ^a^

In order to explore how the observed histopathological changes in the jejunum due to 5-FU induced intestinal toxicity manifested clinically, we assessed diarrhea as a surrogate marker of gastrointestinal mucositis. The control group (A) exhibited no obvious diarrheal symptoms throughout the entire experiment (Fig. 2k). In contrast, group B (5-FU) developed diarrhea from day 10, peaking at day 14 (Fig. 2k), when 55.5% (5 out of 9) of mice had developed mild diarrhea (Fig. 2l), with an overall diarrhea incidence of 77.8% (7/9). Stool consistency score was also significantly increased (*P* < 0.01, Fig. 2k) in the 5-FU treated group (B) compared to control group (A). A chi-square test of independence was performed to examine the relation between 5-FU treatment and the development of diarrhea in mice, which showed a strong significant association between the presence of diarrhea and 5-FU treatment, (chi-square test; *X*^2^(1) = 11.45, *p* < 0.001, Cramer’s *V* test: *V* = 0.798). This result implies that the mice in the 5-FU group (B) had a higher risk of developing diarrhea compared to the control group (A). In general, our morphometric analyses of mouse intestinal tissues demonstrate that 5-FU administration resulted in severe intestinal pathology, disrupting the intestinal epithelial architecture, and this correlated with the onset of diarrhea and clinical manifestations of mucositis.

### Live clinical assessment of chemotherapy-induced oral mucositis

**Figure 3:**
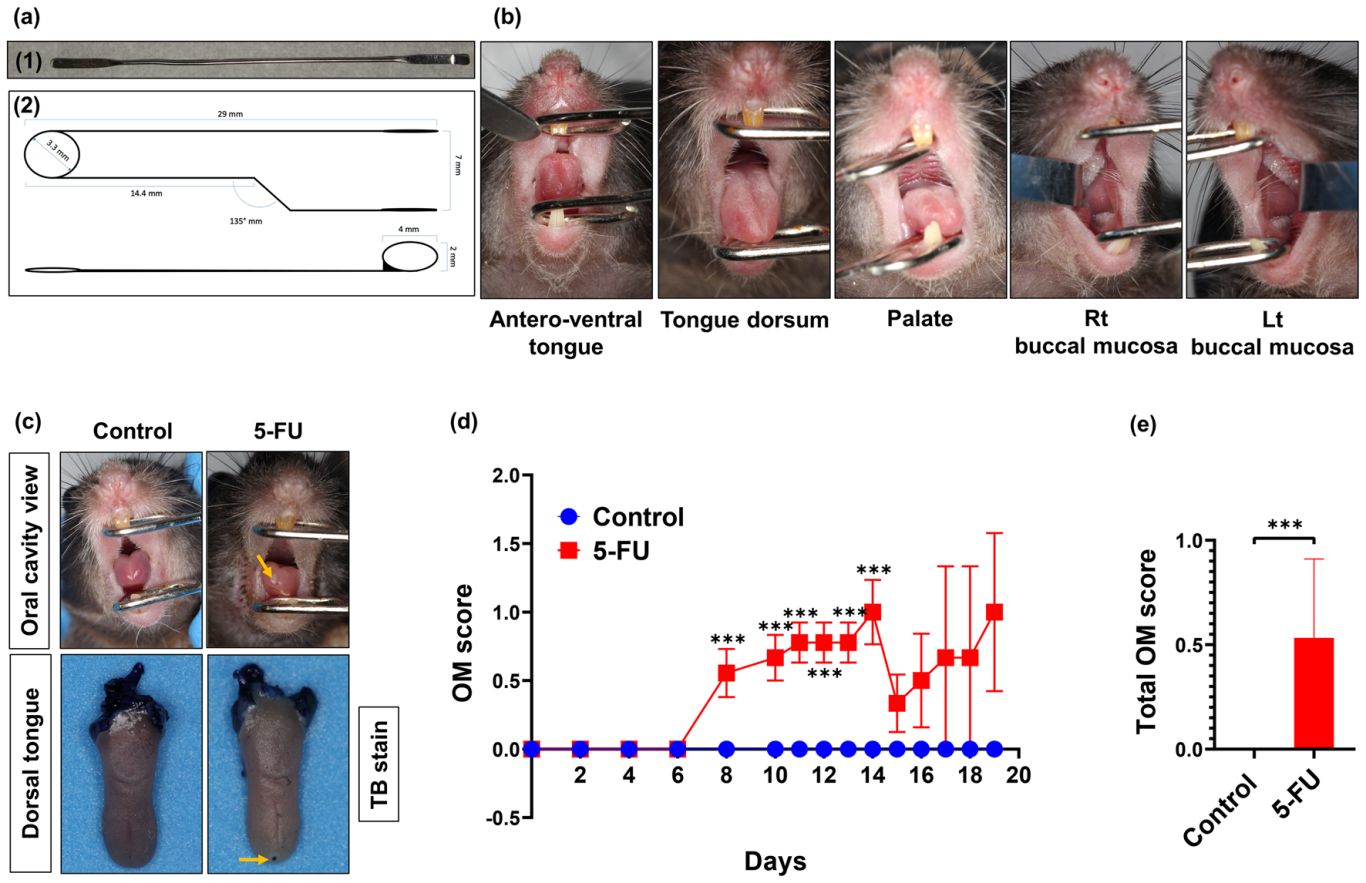
5-FU treatment in mice induces chemotherapy-induced oral mucositis (CIOM). 5-FU (50 mg/kg/day) was injected intravenously to group B at 2-days intervals, starting from day 1 to day 13. Group A (control) injected with saline. Oral mucositis (OM) was scored on a six-point scale as described in Methods. (**a**) Oral cavity examination tools; **1:** Stainless steel laboratory micro spatula; **2:** Schematic diagram of Celentano Murine Mouth Opener (CMMO). (**b**) Oral cavity examination views. Animals were sacrificed at different time points, and tongues collected. Tongues were stained with 1% toluidine blue (TB) in 10% acetic acid. (**c**) Representative photographs show oral mucosa with diffuse erythema on the antero-dorsal surface of the tongue in treated mice, with representative tongues after TB staining showing the epithelium ulceration (blue) at the dorsal surface of the tongue. Yellow arrows show areas of erythema, erosion, and ulceration. (**d**) Comparative OM scores, error bars = mean ± SEM. ***P < 0.001 versus control group. Multiple *t* tests were used to determine the statistical significance between the two groups. (**e**) Mean value of the total score of OM between day-0 and day-19 for each group. Unpaired student *t* test was used to determine the statistical significance between groups. Data are the mean ± SEM (in = 9 for group A and B). ***p < 0.0001.

In order to assess the development and onset of clinical signs of OM, the oral cavity was visually inspected in live animals under general anaesthesia for mucositis/ulcer formation and general appearance by macroscopic means, according to our scoring system (Table 2), using specialized tailored oral cavity diagnostic tools (Fig. 3a, b). In agreement with a previous study^43^, we showed that intravenous (i.v.) administration of 5-FU induced OM development with surface erosive or ulcerative lesions, while OM was absent in the control group (A) (Fig. 3c,d). Clinically, mild OM was first observed in 55.5% of 5-FU recipient mice at day 8 (Fig. 3d). From day 8, mucositis continued to increase in severity, reaching a peak at day 14 (OM score 1.0 ± 0.707, Fig. 3d), with ulcerative lesions (Fig. 3c) followed by further fluctuations in severity grade over time until termination of the study (OM score of 1.0 ± 1.0, Fig. 3d). In summary, our clinical evaluation through OM scores (days 0–19, Fig. 3e), indicated a clinical manifestation incidence of 88.98% in the 5-FU treated mice (*p* < 0.0001).

**Table 2:** Visual oral ulcerative mucositis score^118^: based on a modification of the method of Sonis et al.^119^.

Grade	Severity	Description
0		Normal (no abnormalities)
1	Mild	Partial hyperemia, erythema and swelling, with no evidence of mucosal erosion (mucosa intact)
2	Mild	Overall hyperemia, erythema and swelling and superficial erosion, may include mild sloughing
3	Moderate	Epidermolysis, hyperemia and erythema; moderate mucositis characterized by frank ulcer formation. Ulcers typically have areas of necrosis (erosion) with associated yellowish/grey coloration with pseudomembrane formation
		Cumulative area of ulceration ≤ 25% of tongue surface area
4	Severe	Extensive epidermolysis and bleeding; severe mucositis with ulcer formation affecting 25–50% of tongue or buccal mucosal area. Marked erythema and pseudomembrane formation. Loss of pliability
5	Severe	Bleeding and abscesses; virtually complete ulceration of the mucosa, all mucosa areas at risk. Loss of mobility and pliability

### Histological assessment of experimental oral mucositis

**Figure 4:**
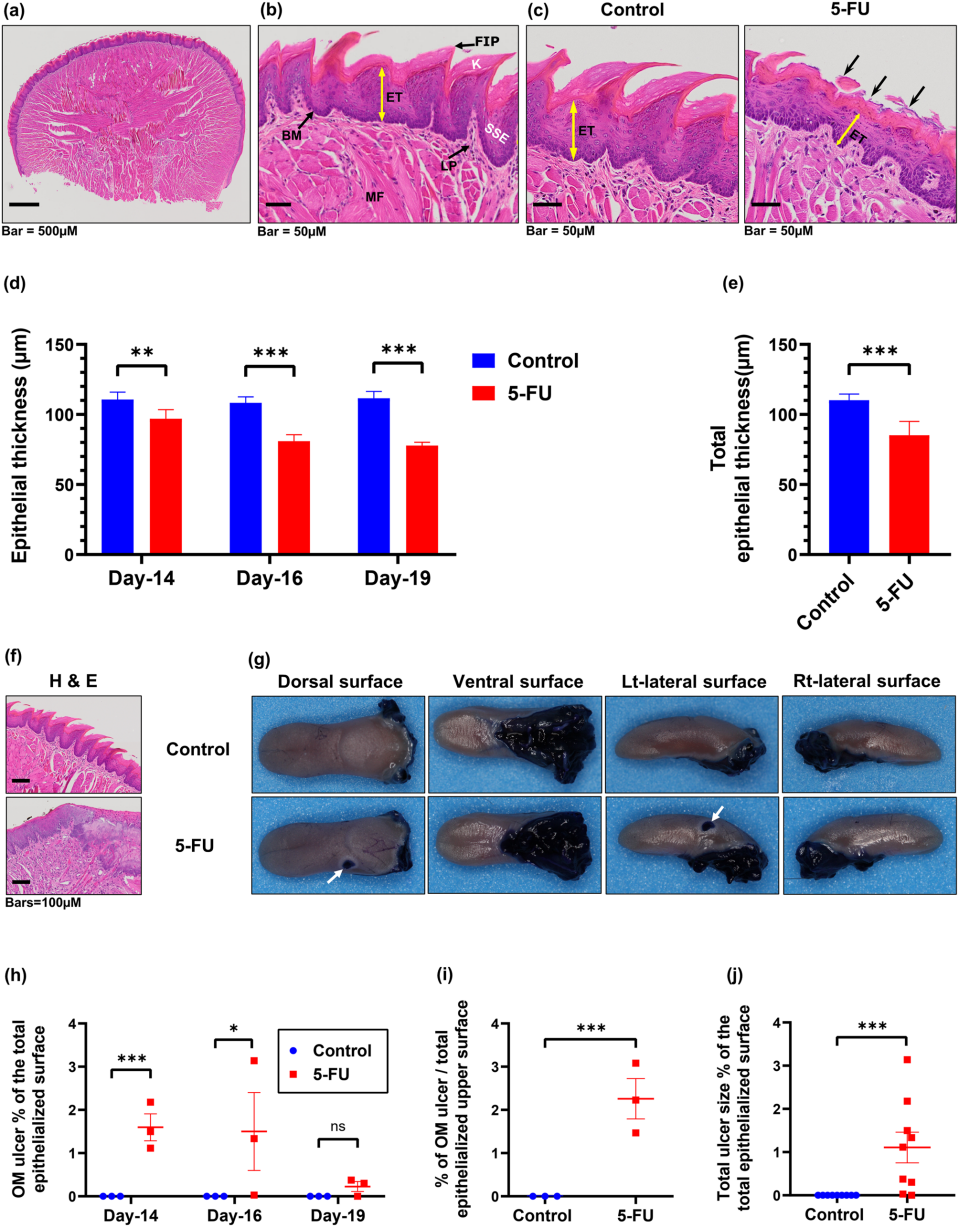
5-FU-induces tongue mucosa damage and oral mucositis in mice. Histological changes of the tongue were determined by H&E staining. (**a**) Representative photomicrograph showing the histologic section of the tongue base from control animal. (**b**) Representative photomicrograph of dorsal tongue mucosa showing keratin layer (K), filiform papillae (FIP), stratified squamous keratinized epithelium (SSE), basement membrane (BM), lamina propria (LP), and muscle fibers (MF), with epithelial thickness (ET) measured from basal membrane to epithelial granular layer. (**c**) Representative photomicrograph of the tongue mucosa from control and 5-FU-treated mice. Compared with control mice, the superficial regions were largely destroyed, and the epithelial thickness (double headed yellow arrow) was reduced (epithelial atrophy), with the filiform papilla being entirely damaged (black arrows). Identical histological aspects were confirmed in multiple control, and 5-FU-treated mice. (**d**) Graphical representation of the average epithelial thickness of the dorsal tongue at each time point measured. Unpaired student *t* test was used to determine the statistical significance between groups. (**e**) Graphical representation of total epithelial thickness of the dorsal tongue between Day-14 and Day-19. Multiple *t* tests were used to determine the statistical significance between the two groups. Data represent Mean ± SD (18 representative measurement/tissue; three representative mice/group/timepoint).**p < 0.005. ***p < 0.001. (**f**) Representative photomicrograph shows tongue sections from group A and B, with the connective tissue in the 5-FU group (A) showing cellular infiltrate indicative of an inflammatory response. Black arrow shows area of erosion. (**g**) Representative photographs show TB stained tongues revealing CIOM ulcers (blue staind, weight arrows) at various tongue surfaces. Pearson chi-square tests for independence (categorical variables) were used to examine differences in ulcer status Tongues were stained with 1% TB in 10% acetic acid, imaged, and the ulcer size and the total epithelialized surface of the tongue were measured. (**h**) Graphical representation of the mean oral mucositis (OM) ulcer size as a percentage of the total epithelialized surface of the tongue (days 14, 16, and 19). Multiple *t* tests was used to determine the statistical significance between the two groups. (**i**) Graphical representation of the mean percentage OM ulcer size compared to the total epithelialized upper surface of the tongue on day-14. Unpaired student *t* test was used to determine the statistical significance between groups. (**j**) Graphical representation of the total mean OM ulcer size as a percentage of the total epithelialized surface of the tongue between day-14 and day-19. Unpaired student *t* test was used to determine the statistical significance between groups. Data represent the mean ± SEM (*n* = 9, and 9 for group A and B, respectively). *p < 0.05; ***p < 0.001.

Histologically, the normal stratified squamous keratinized epithelium of the human and murine tongue consists of several layers of polyhedral cells, flattening to scales and keratin which ultimately shed from the surface^44,45^. The dorsal surface of the tongue also has hair-like filiform papillae (FIP) formed by epithelium and keratin layers, responsible for the sensation of touch (Fig. 4a, b).

In order to explore the effects of 5-FU treatment on the histological structure, and thus functionality, of the tongue we assessed H&E sections on days 14, 16, and 19 harvested from both control and 5-FU treated mice. Epithelial thickness of the tongue was measured as described by Carrard et al.^46^ from the basal membrane to the granular layer (Fig. 4a, b). Our analysis showed that between days 14 and 19, the normal murine tongue morphology in the control group (A) was preserved with no visible lesions (Fig. 4c). In contrast, 5-FU administration resulted in epithelial atrophy, mirrored by a significant reduction in epithelial thickness compared to the control group (A) (Fig. 4c,d,e). In addition, 5-FU treatment affected the structural integrity of the dorsal mucosa resulting in complete destruction of the filiform papilla and a change in appearance of the keratinized layer from uniform to desquamative (Fig. 4c). Further severe changes observed, included breakdown of the entire epithelia leading to ulcerative lesions covered by a thin basal layer of necrotic fibrinoid material, margins with inflammatory cell infiltrating the affected regions, and active granulation (Fig. 4f). In conclusion, 5-FU induces severe histological damage in the tongue, likely also resulting in functional impairment.

In addition to the clinically visible oral complications of 5-FU (Fig. 4c), this treatment results in sustained partial or complete loss of the tongue epithelium in humans^47,48^. In order to explore this complication more thoroughly in our murine model, we conducted an in-depth chemotherapy-induced ulceration assessment post-mortem using TB staining. This demonstrated that OM ulceration was localized mainly to the tongue dorsolateral and ventral surfaces (Fig. 4g), with TB positive areas of Chemotherapy-induced oral mucositis (CIOM) (Fig. 4g, white arrows) corresponding to erosive or ulcerative lesions with either extremely atrophic or absent epithelium (Fig. 4f, group B). Ulcerated lesions were covered by necrotic fibrinoid tissue with complete loss of the stratified squamous keratinized epithelium accompanied by an inflammatory cell infiltrate. TB-stained tongue CIOM ulceration was present in 88.89% of animals in the 5-FU treated group (B) and absent in the control group (A). Quantitative analysis of the dorsal and ventral tongue surfaces showed that 5-FU treated animals (group B) had significantly larger ulcer mean size percentage of the total epithelial surface at day-14, (1.597 ± 0.31%, *t* test: *p* < 0.001, Fig. 4h), which subsequently declined. At this timepoint, the CIOM ulcer size percentage compared to the total epithelialized upper surface of the tongue peaked in the 5-FU treated mice (2.259 ± 0.81%, Fig. 4i, j) and was significantly elevated compared to control group (A) (unpaired *t* test: *p* < 0.001). Our analysis demonstrates that 5-FU treatment in our murine model induces clinical effects consistent with chemotherapy-induced OM.

### 5-FU administration triggers an inflammatory response in mice

**Figure 5:**
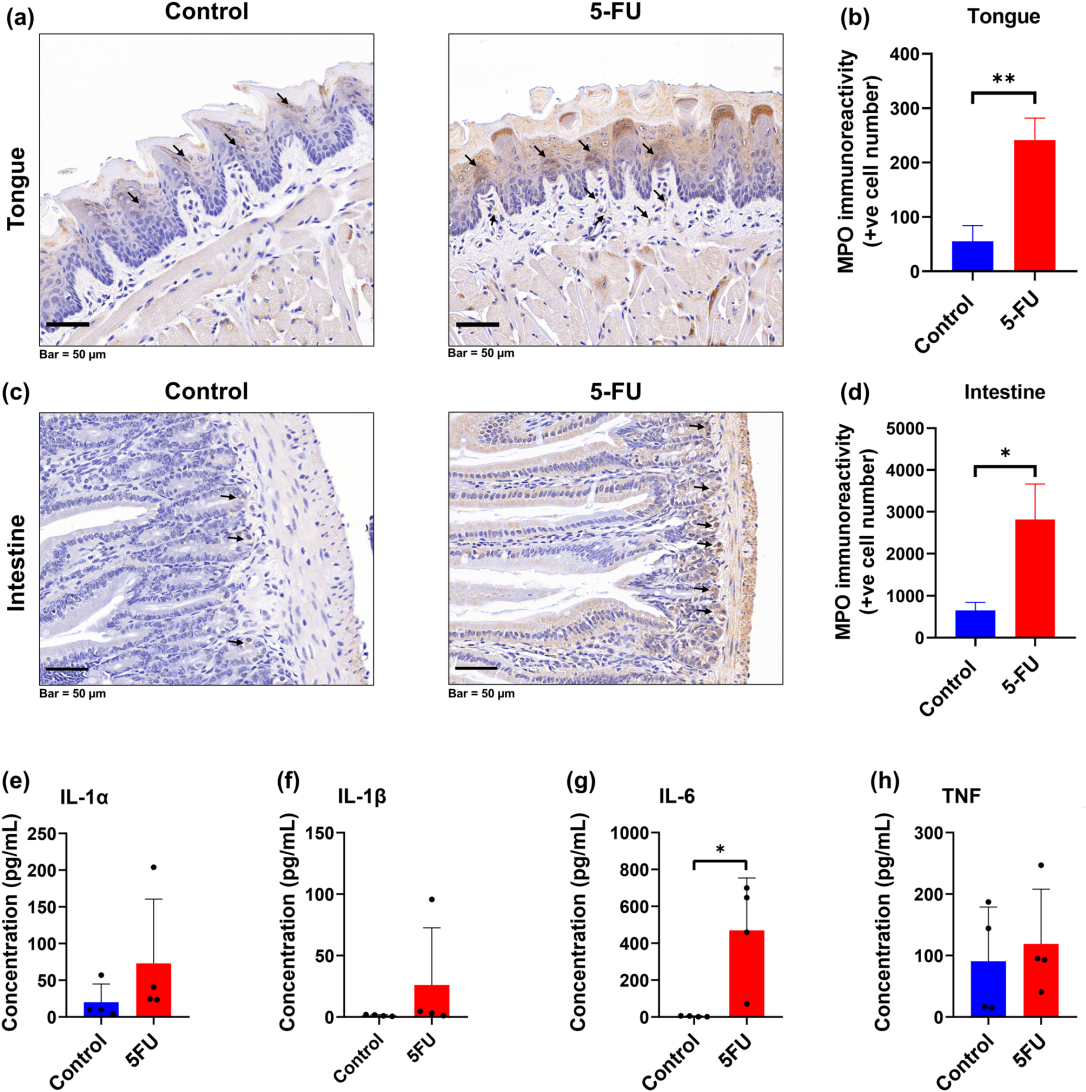
Effect of 5-FU on MPO levels in tongue and jejunum tissues, and inflammatory cytokynes levels in serum. Immunohistochemical analysis for MPO expression. Mice received 5-FU Intravenously (i.v.), withtongue and jejunum were excised at sacrifice on day 14. MPO immunolabelled cells in tongue and intestinal mucosal sections were counted. MPO positive cells were counted in tongue mucosa of each section from three different transverse sections/tongue of three representative mice/group. MPO positive cells in the intestine were counted from twelve full villus per jejunum section, four intestinal sections per mice, with three representative mice per group. (**a**) Representative microphotograph showing immunohistochemical staining for MPO in tongue mucosa of C57BL/6 mice (images at magnification X20). MPO positive cells were detected in the epithelial and lamina propria area (black arrows; bar indicates 50 μm). (**b**) Graphical representation of the number of MPO positive cells/tongue. Data are expressed as mean ± SD. (**c**) Representative microphotograph showing immunohistochemical staining for MPO in small intestine of C57BL/6 mice (images at magnification X20). MPO positive cells were detected in the tunica mucosa and submucosa area (black arrows; bar indicates 50 μm). (**d**) Graphical representation of the number of MPO positive cells/intestine. Data are expressed as mean ± SD. Levels of pro-Inflammatory cytokine proteins, IL-1α (**e**), IL-1β (**f**), IL-6 (**g**), and TNF (**h**), were determined in serum at day 14. Data are expressed as mean ± SD. *P < 0.05, **P < 0.005.

The extent of the neutrophil accumulation in the tongue mucosa and jejunum tissue samples was measured by the quantification of myeloperoxidase (MPO) activity, a neutrophil enzyme marker. Immunohistochemical analysis indicated that 5-FU administration resulted in a more than threefold increase in MPO-positive cell number in tongue (*p* < 0.005) and intestinal (*p* < 0.05) mucosa on day-14 compared to control animals (Fig. 5 a,b,c,d, arrow heads).

Interestingly, the inflammatory response in the tongue mucosa and jejunum due to i.v. 5-FU treatment was associated with a systemic changes reflected as an elevation in the levels of serum pro-inflammatory mediators, including Interleukin-1α (IL-1α), IL-1β, IL-6 and tumour necrosis factor (TNF) (Fig. 5e,f,g,h). These inflammatory markers are strongly associated with oral and oral and gastrointestinal mucositis in humans^9,49^. In particular, day 14 of i.v. 5-FU treatment was associated with a significant increase in serum level of IL-6 (Fig. 5g). This suggests that i.v. chemotherapy protocols trigger a significant pro-inflammatory response in oral and intestinal tissues.

## Discussion

Mucosal inflammation and atrophy of the mouth and gastrointestinal tract, collectively known as alimentary tract mucositis, are debilitating adverse effects observed in cancer patients undergoing chemotherapy^50–52^. In the present study, we used i.v. administration of 5-FU to induce simultaneous oral and intestinal mucositis in mice and characterized a mouse model of mucositis that replicates the features observed in both oral and gastrointestinal tract mucositis. Furthermore, we undertook a thorough clinical and histological assessment of the oral cavity that can be used as a reference for pre-clinical research. While mechanical-, chemical- or radiation-induced injury of the oral mucosal surface is additionally required in most existing CIOM models^24,53^, our model is a significant improvement over such models because it: (i) does not require additional oral mucosal “irritation” to induce macroscopically and histologically recognizable oral erosions and ulcers; (ii) reproduces the clinical and histologic features of both oral and intestinal mucositis induced by chemotherapy. (iii) triggers a significant clinical ulcerative response in oral epithelium without severe loss of intestinal function; and (iv) resembles most of the current human anti-cancer 5-FU regimens^54^.

In our study, 5-FU was used as a prototype chemotherapeutic drug, since it is one of the foremost effective chemotherapeutic agents used in the treatment of advanced colorectal cancer and malignancies of the head and neck^55,56^. However, its clinical use is limited by the severity of the side effects, including CIOM and diarrhea^56,57^. Despite various available treatment options to alleviate these complications, there remains a necessary and urgent need for better effective therapies to alleviate or even prevent CIOM. Such therapies may only be achieved with comprehensive characterization and documentation of a pre-clinical animal model. While several pre-clinical models of CIM do exist and studies have extensively reported that the administration of 5-FU to experimental animals causes both oral and intestinal mucositis^43,58–60^, research has mostly focused on either oral or intestinal mucosal damage^31–36^ using diverse experimental designs of mucositis models in rats^33^, hamsters^61^, and mice^25,62,63^. One of the first animal models of chemotherapy-induced oral mucositis was described by Sonis and colleagues (1990)^61^, who combined 5-FU intraperitoneal (i.p.) injection with mechanical irritation using hamsters. However, this model studied only the oral mucosal damage associated with the chemotherapy. In addition, the combination of mechanical irritation with chemotherapy raises controversy as to whether this may be a chemotherapy-induced impaired wound healing model, where chemotherapy's direct effect on the healthy epithelium cannot be detected or measured. Pertinently, most of the existing CIOM rodent models are not systemic in nature, following administration protocols, which do not significantly affect the oral mucosa unless local mechanical or chemical injury are included^31–33^. For example, in two different studies of CIOM murine models^31,32^, while successful in the generation of obvious ulcerated OM lesions, these were not exclusively induced by 5-FU but by the supplementation of chemical stimuli (20% acetic acid). Furthermore, these studies lacked vital histopathologic assessment, which represents the foundation for the current understanding of the pathophysiology of CIOM^64^. In another study of CIOM in rats^33^, administering daily triple i.p. injections of 5-FU at 5-days intervals, together with superficial needle scratching of the oral mucosa, resulted in OM development. This included damage to the buccal mucosa, ulceration, and abscess formation, together with loss of collagen bundles and inflammatory cell infiltration, confirmed by histopathology^33^. While this latter model reproduces the histopathological and inflammatory responses of the 5-FU-induced oral mucositis, mechanical injury of the mucosal surface was required to induce ulceration on buccal mucosa^53^. In addition to 5-FU-induced mucositis murine models, a series of rodent models with different cancer treatment regimens (drugs, dosage, treatment duration and follow-up period) have been developed^23,65,66^. CIM murine models have evolved for various chemotherapeutic agents such as irinotecan^67,68^, MTX^34^, cyclophosphamide^69^, 5-FU^70^, cisplatin^71^, melphalan^72^, doxorubicin^73^, cytosine arabinoside (AraC), and vincristine^74^, as well as combinations of irinotecan and 5-FU^75^ among others. Study of various CIOM animal models have shown that these chemotherapies were each insufficient to induce oral mucositis^43,70,76^, unless accompanied by local mechanical or chemical injury^24,29,30^. Given that, most of the existing CIM murine models focus on evaluating the mucosal changes of the lower gastro-intestinal tract^27,35,36,66^, these studies preclude the study of both oral and intestinal mucosal damage within the same experimental model. Therefore, these protocols deviate from the real pathological clinical scenario faced by the great majority of cancer patients. Our study is distinctive, in that it is the first murine model of intravenous chemotherapy-induced oral and intestinal mucositis reproducing the clinical and histopathological responses of both oral and intestinal human mucosa to 5-FU resembling most of the current human anti-cancer 5-FU regimens.

Clinically, the manifestations of OM form a continuum; mucositis starts in an asymptomatic manner, followed by the presence of erythema, complaints of burning, and increased sensitivity to hot and spicy foods. The erythema may progress to areas of desquamation, followed by the appearance of large areas of deep, coalescing painful ulcers^77,78^. These are characterized by an irregular shape and a poorly defined borderline erythema. The most frequently affected areas are nonkeratinized mucosae of the labial and buccal mucosa, dorsal, lateral, and ventral surface of the tongue, soft palate, and floor of the mouth^22,79^. In the present study, we attempted to capture all such variables by undertaking a comprehensive oral cavity examination. The results presented herein are consistent with the previous study demonstrating that i.v. administration of 5-FU triggered diffuse erythema on the dorsal surface of the tongue, with the toluidine blue positive areas corresponding to erosive or ulcerative lesions^43^. The histopathological evaluation of CIOM presented here clearly establish correlations between the clinical and pathological assessment with those observed in human oral mucositis^6,80^, thus supporting this mouse model as ideal for pre-clinical studies.

It has been clearly demonstrated in experimental animals, that 5-FU-induced diarrhea and BW loss are accompanied by morphological damage to the small intestine^81–83^, with IM morphologically characterized by the shortening of villus height and destruction of crypts in the small intestine^84^. Further, an essential feature of 5-FU-induced mucositis is inflammation and atrophy of both the oral and intestinal mucosa accompanied by leukocyte infiltration^85^. We clearly demonstrate major histological damage of the jejunum resulting in shortening of villi length, distortion of crypts, excessive inflammatory cell infiltration and cellular damage in mice after 5-FU administration. Our findings are consistent with those of Bertolini et al.^43^, who also observed significant atrophy of the oral and jejunum epithelium in a murine model of 5-FU-induced mucositis. However, this previous study did not investigate the role of 5-FU in diarrhea incidence and severity in the gut. Our present findings show that repeated i.v. administration of 5-FU results in severe IM in mice, are consistent and extend this to the measured clinical effects^27^. During the inflammatory phase of mucositis, epithelial damage and inflammatory cell infiltration in the murine mucosa have been notably reported in the previous studies^7^, with a significant increase in villous atrophy in the intestinal mucosa after 5-FU treatment^86^.

Body weight loss and diarrhea are common side effects of patients treated with 5-FU^87,88^. While these complications are clinically relevant aspects in CIM experimental animal model studies^35,89^, they are challenging due to dose- and time-dependent morbidity and mortality^35,89^. Several studies have shown that 5-FU produces diarrhea and BW loss accompanied by an increase in mortality in experimental animals^35,89,90^. In agreement with these studies and others^91,92^, we report that the 5-FU induces significant weight loss and moderate diarrhea, with accompanying low mortality. Notably, weight loss is a common clinical feature and frequent for concern in bowel cancer patients, originating from many factors, including diarrhea, nausea, and intestinal toxicity^93^. Diarrhea is one of the most common adverse reactions to 5-FU, which can occasionally lead to discontinuation of treatment, hospitalization, and other severe symptoms, including death^94^. To determine whether these adverse effects were associated with mucositis in our mice model, these variables were compared in 5-FU treated mice. A close correlation between severity, degree, and duration of the chemotherapy-induced OM and IM, diarrhea score and severity, and animal BW loss was observed, peaking days 14–16 and associated with IM severity^81^. BW reduction could be partially explained by the physical obstruction of food and fluid intake due to ulceration with diarrhea, and the systemic inflammatory reaction likely to further exacerbate weight loss^27^.

At a biomolecular level, MPO activity can chart the inflammatory process and oxidative stress occurring in the chemotherapy-damaged mucosal tissues. MPO is the most common proinflammatory enzyme and is stored in the azurophilic granules of neutrophilic granulocytes^95^. Neutrophil infiltration is a key feature of acute inflammation^43,96^. Therefore, measurement of the MPO enzyme levels provides a reliable marker of neutrophil infiltration and hence acute inflammation^97^. Furthermore, among the variety of reactive oxygen species generated in tissues in response to radiation, hypochlorous acid (HOCl) is formed from hydrogen peroxide via catalysis induced by myeloperoxidase (MPO) secreted from neutrophils^98^. It also catalyzes oxidation of thiocyanate (SCN^−^) to generate another ROS, hypothiocyanite (OSCN^−^) via a similar reaction^99^. In the present study, we compared the MPO activity in the tongue and jejunum tissues of 5-FU treated and control mice. The assessment revealed that normal tissue contain low levels of MPO. Several previous studies have also shown similar low levels of MPO exists in normal tissue^100,101^.

Given the damage in the oral and intestinal mucosa caused by chemotherapeutic agents, an increase in MPO levels would be expected because of concomitant inflammation. In fact, studies of mucositis in multiple animal models support this hypothesis^102–105^. Nevertheless, studies by Keefe and co-authors^40^ suggeststhat the role of inflammation in the progression of mucositis remains to be determined.In the current study, an increase in MPO activity was detected in both the tongue and jejunum in the i.v. 5-FU-treated groups, implying that inflammation was present. Regarding its activity, MPO is a key factor in a variety of immune-mediated inflammatory conditions, affecting but not limited to the kidney, and brain^95^. Therefore, MPO and its downstream inflammatory pathways might be attractive targets for both prognostic and therapeutic intervention in the prophylaxis of chemoradiotherapy-induced mucositis.

Interestingly, the inflammatory response in the tongue mucosa and jejunum due to i.v. 5-FU treatment was associated with an increased serum levels of pro-inflammatory cytokines, including IL-1α , IL-1β, IL-6 and TNF. Chemotherapeutic agents, including 5-FU cause upregulation of stress response genes, including the NF-κB p65/RELA subunit, leading to tissue injury by increasing the production of proinflammatory cytokines such as IL-1β, IL-6 and TNF. It is well known that inflammatory cytokines, particularly IL-1β, IL-1α, IL-6 and TNF, play important roles in the pathophysiology of oral mucositis^9,49^. The inflammatory phase of chemotherapy-induced mucositis is initiated by a disruption in DNA synthesis, which alters metabolism in the progenitor cells of rapidly dividing epithelia. This can lead to inhibition of mitosis, disruption of cell–cell and cell-substrate interactions, and a decreased epithelial integrity. Following these events, proinflammatory cytokine levels rise, followed by neutrophil infiltration^106^. The development of this mouse model allows for further mechanistic studies on the regulatory networks of mucosal inflammation and apoptosis induced by 5-FU and the means to test the contributions of other signaling pathways such as oxidative stress and NF-κB in each biological process.

While our model did not account for the role of oral and gut microbiota, which may have influenced the response to 5-FU and/or to other drugs tested, nevertheless mice developed clinical and histopathological features consistent with human disease. As with most CIM research, the main focus of our work was the assessment of mucosal effects of chemotherapy regimens in healthy animals, independently of interactive tumor effects, since this allows for a more direct interpretation of specific effects of chemotherapy^66^. In addition, tumors implanted in rodents often involve a larger fraction of the total body weight than in humans, leading to obvious effects on the whole body^107^. This aspect complicates the translation of CIM findings in the models of tumor-bearing rodents, which may explain some of the discrepancies between animal and human studies in terms of studying nutrition, chemotherapy, and tumor growth^108,109^. Another element to consider with 5-FU toxicity in mice is the relatively high drop-out rates (25%). Due to ethical reasons, all animals undergoing a BW reduction > 20% before the last dose of 5-FU treatment were necessarily withdrawn and were not included in the final statistical analysis, (animal ethical consideration). Another limitation is that we focused on the histological effects of 5-FU on the jejunum part of the small intestines; other parts of the intestine, such as the duodenum and ileum, or gastrointestinal tract, such as colon and stomach specimens, were not examined. However, literature has shown that the jejunum is the most severely damaged portion of the intestine after several chemotherapeutic regimens^110–112^. The inability to monitor individual food and water intake since mice were housed in groups, is an additional limitation to this study. Notwithstanding these limitations, the present study provides the most thorough murine clinical-pathological assessment of alimentary tract mucositis associated with 5-FU to date. We propose that this model can be used to inform the development and testing of novel preventative or therapeutic strategies for this most debilitating condition.

In summary, in the current study, i.v. administration of 5-FU in mice resulted in oral epithelial layer loss, ulcerative OM, moderate diarrhea, bodyweight loss, damaged intestinal mucosa, villi lengths shortening, and loss of crypt architecture in the jejunum. OM commenced in mice from day-8, with the TB-stained CIOM ulceration on the tongues evident and subsequent OM scoring reaching a maximal at day-14. Furthermore, we demonstrate weight fluctuation in relation to chemotherapy-induced oral and intestinal mucositis and found a relationship between OM severity and score, diarrhea severity, and weight loss. Since the onset of murine oral and intestinal mucositis manifests profound similarities with the onset of human oral and intestinal mucositis observed after chemotherapy^6,93^, we consider that this model is a suitable in vivo pre-clinical model of chemotherapy-induced alimentary tract mucositis.

## Materials and methods

### Animal experiments

C57BL/6 female mice aged 6–12 weeks and weighing between 15 and 20 g were used in this study. Animals were purchased from Bio21 animal facility (Bio21 institute, The University of Melbourne, Parkville, Victoria, Australia). Mice were housed in groups of three or four in clear cages with wood chips under specific pathogen-free conditions and maintained on a 12 h light/dark cycle in a temperature-controlled and humidity-controlled room (21 ± 1 000bC and 40–60%, respectively) with free access to autoclaved food pellets and water provided ad libitum. To investigate the adverse effects associated with the administration of 5-FU, mice were randomly divided into two groups, A (control, *n* = 9) and B (5-FU, *n* = 12). Animals were sacrificed on days 14, 16, and 19, in order to monitor, both macroscopically and microscopically, incidence, development, and extent of mucositis/ulcers (including severity, degree, duration, and healing) over time. This study was approved by the Animal Ethics Committee of The University of Melbourne (ID: 1,814,636.3). All work was conducted in compliance with the Australian Code for the Care and Use of Animals for Scientific Purposes determined by the National Health and Medical Research Council 8th edition (2013)^113^. And reported according to the ARRIVE guidelines.

### Induction of chemotherapy induced oral and intestinal mucositis (CIOIM)

5-FU powder (Sigma-Aldrich, Castle Hill, NSW, Australia Cat#F6627) was dissolved in saline to a final concentration of 10 mg/ml. Mice were induced to develop mucositis as previously described^43^. Briefly, a total of 21 mice were used in this study, randomly divided into two experimental groups, namely group A (injected with saline, *n* = 9) and group B (injected with 5-FU, *n* = 12) (Fig. 1a). Mice received a single intravenous (i.v.) injection of 5-FU (50 mg/kg/day) or saline (5 ml/kg/day) every 48 h, from days 1–13. The dose and frequency of 5-FU administration were chosen based on a previously published study, as these were effective in substantially reducing tumor size in a xenograft mouse model^114^. Furthermore, the dose is within the therapeutic intermittent dose range in humans^115,116^, and comparable to infusion doses recently used for the treatment of head and neck cancer patients (approximately 800 mg/m^2^)^117^. Mice were monitored daily for signs of morbidity, and their body weight (BW) recorded every 24–48 h.

### Assessment of weight, diarrhea, and fatalities

Over the 19-days observation period, mice were monitored daily for signs of morbidity, and the bodyweight of each animal was recorded at the beginning of the study and every 48 h post initial injection until day 10, then every 24 h until the end of the experiment. Visual scoring included: clinical records for the presence of dull/ruffled coats, temperament changes (squealing when handled, stress marks on paws/face, lethargy). At onset of weight loss (grade 2 OM index or > 5% weight loss), a semi-solid diet was promptly instituted.

The severity of 5-FU-induced gastrointestinal mucositis was assessed by scoring of stool passages (diarrhea assessment) every 48 h post initial injection until day 10, then every 24 h until the end of the experiment. Diarrhea severity was assessed by using Bowen’s score system^68^, and classified into four grades: 0, normal stool; 1, slightly wet and soft stool (mild diarrhea); 2, wet and unformed stool (moderate diarrhea); 3, watery stool (severe diarrhea).

5-FU dose was reduced to 50% when weight loss exceeded 15%. Mice exceeding 20% of BW loss were sacrificed immediately and included in fatality count. Animals reporting a BW loss > 20% before the last dose of 5-FU treatment were withdrawn from the study and not included in the final statistical analysis, following a “per protocol” approach. Post last dose of 5-FU treatment, remaining mice were euthanised on days 14, 16, and 19.

### Assessment of oral mucositis

Starting from day 2 (one day post initial injection) mice were anesthetized with isoflurane every 48 h post initial injection until day 10, then every 24 h until the end of the experiment. Macroscopic oral cavity inspection, including anteroventral and dorsal tongue, palate, floor of the mouth, lips, and right and left buccal mucosa, were examined for mucositis/ulcer formation and general appearance using specialized tailored oral cavity diagnostic tools. Incidence, development, and extent of OM were assessed clinically in vivo by using the visual oral ulcerative mucositis score, adapted from Nakajima et al.^118^, based on a modification of the methods of Sonis et al.^119^ (Table 2). Image acquisition was recorded digitally (Canon EOS 60D; Canon Inc., Tokyo, Japan) using 100 mm lens (Canon 100 mm f/2.8 Macro USM lens) connected with a ring flash (Sigma EM-140 DG Ring Flash).

### Anaesthesia and euthanasia

For oral mucositis index scoring, mice were anesthetized with isoflurane every 24–48 h. Mice induction was briefly done in a chamber at 5% isoflurane in oxygen until the loss of the righting reflex occurred. Animals were then left in the chamber with isoflurane 2% in oxygen for 2–3 min before being removed to perform the oral mucositis scoring. The time required for mice examination and image collection for each mouse was approximately 2–3 min.

At the end of each experimental time point (specifically days, 14, 16, and 19 of the experiment), and after weighing the animals, mice were euthanized by exsanguination via cardiac puncture under general anaesthesia. Briefly, general anaesthesia was induced in a chamber at 5% isoflurane in oxygen until loss of the righting reflex occurred. Animals were then maintained on 2–3% isoflurane via a nose cone and laid on the back, and a 25–26 gouge needle was inserted into the heart at a 15–30° angle from the point just beyond the sternum. Negative pressure was applied slowly to withdraw the maximum amount of blood (~ 1 ml) from the heart. To ensure the mice were dead, cervical dislocation was also performed.

The general anaesthesia procedure and post procedure monitoring were conducted in accordance with the Melbourne University’s “General anaesthesia for mice and rats” Animal Care and Use Standard.

### Macroscopic examination of the tongue

At necropsy, the tongue was excised at the level of the trachea. To neatly reveal surface erosive or ulcerative lesions, the tongue was stained with 1% toluidine blue (TB) in 10% acetic acid for 1 min, followed by repeated washes with 1% acetic acid until no further recovery of dye^120^. Tongue images were acquired with a digital camera as for assessment of OM and scored according to the following criteria: Negative—lack of dye uptake or light, diffusely stippled uptake of dye; positive—deep, royal blue staining of the epithelium and lack of epithelium (identified as ulcer). The percentage of TB positive surface area (excluding excision trauma) was calculated using Fiji (ImageJ) software^121,122^.

The assessment of murine body weight, condition, appearance and behavior, diarrhea, fatalities and evaluation of oral mucositis were carried out by two clinicians (AC and AM) and one expert scientist (RP). All examiners were competent and trained in handling, examining, and scoring small laboratory animals. The assessment was calibrated and scored and the agreed between examiners.

### Histopathologic examination

At necropsy, jejunum and tongue specimens were collected and fixed in 10% neutralized formalin overnight. For the jejunum specimen collection, the stomach was identified and traced to where it joins with the small intestine. With a pair of scissors, the intestine was cut about 1 cm from the stomach. As there is no anatomical limit defined between the duodenum, the jejunum, and the ileum for obtaining jejunum samples, the middle of the small intestine was considered during sample collection^123,124^. The small intestine was held with one hand or a pair of forceps, and an approximately 6–10 cm section cut of the proximal jejunum (located nearer to the stomach).

The tissues were embedded in paraffin, cut into 4-µm-thick sections, and stained with hematoxylin and eosin (H&E). Slides were scanned as digital images using Olympus VS120 automated slide scanner equipped with a BX61VS microscope and 20 × objective (Olympus VS120-S6-W, Olympus VS-ASW software). Quantitative histomorphometry measurements of the intestinal wall of the jugum were performed on the scanned digital images of the slides using QuPath open source digital software v. 0.2.0^125^. Morphometric analysis of the intestinal wall of the jejunum required tissue samples to be oriented for longitudinally cut villi, to assess tunica mucosa thickness, intestinal villi length and crypt depth. Analysis was conducted as described by Navarrete et al.^126^. Briefly, on each slide, thickness of the tunica mucosa was determined. In addition, the length of the intestinal villi and depth of intestinal crypts were assessed (measured from the base of the intestinal villi to the lamina muscularis mucosae). Three mice per group were analysed, with the analysis consisting of 3 intestinal ring sections and one longitudinal intestinal section per mouse. Each analysis incorporated 3 fields per section and 1 measurement per field, with twelve intact villi and crypts (full-sized not exhibiting bending or mechanical damage) measured and averaged. For the morphometric analysis of the tongue, the epithelial thickness was measured using QuPath open-source digital software v. 0.2.0^125^, from digital images scanned using Olympus VS120 automated slide scanner at 20X magnification as described by Carrard et al^46^. Briefly, epithelial thickness of the tongue dorsum was measured from the basal membrane to the granular layer. Three mice per group, and two transverse (coronal) sections per tongue, consisting of 3 fields per section (selected at random, considering the complete width of the dorsum) and 3 measurements per field, were measured and averaged. All measurements were conducted by a single observer (AM).

### Immunohistochemical staining

Formalin-Fixed, Paraffin-Embedded (FFPE) tongue and jejunum samples were sectioned onto Superfrost slides (Thermo Fisher Scientific, MA, USA), followed by deparaffinization and rehydrated according to standard protocols. Slides were then prepared for IHC with anti-Myeloperoxidase (MPO) (ab65871, Abcam, Australia) rabbit polyclonal antibody using mouse and rabbit specific HRP/DAB IHC detection kit—micro-polymer (ab236466, Abcam, Australia) according to manufacturer's instructions. Briefly, rehydrated slides were treated with hydrogen peroxide (Abcam, Australia), which was followed by heat-induced epitope-retrieval (HIER) with 0.1 M citrate buffer for 15 min. The sections were then incubated with the protein-blocking reagent (Abcam, Australia) for 10 min and then treated with a specific antibody for MPO (Abcam, Australia; 1:1250; 4 °C for overnight). Post PBS washing, the sections were incubated with goat anti-rabbit IgG secondary antibody (Abcam, Australia) at room temperature for 15 min and developed using HRP-conjugated DAB substrate (Abcam, UK), followed by counter-staining with hematoxylin. Grading was performed based on positive stain counting on the scanned digital images of the slides using QuPath open-source digital software v. 0.2.01^125^.

### Multiplex analysis of serum cytokine levels

Serum cytokine and chemokine levels were measured using the Bio-Plex Pro™ mouse cytokine magnetic bead immunoassay (23-plex; Bio-Rad# M60009RDPD) on a Bio-Rad Bio-Plex instrument, following the manufacturer’s instructions as before^127^. The cytokines measured include L-1α, IL-β, IL-2, IL-3, IL-4, IL-5, IL-6, IL-9, IL-10, IL-12(P40), IL-12(p70), IL-13, IL-17A, Eotaxin, G-CSF, GM-CSF, IFN-γ, KC (CXCL1), MCP-1, MIP-1α, MIP-1β, RANTES and TNF.

### Statistical analyses

Statistical analysis was performed using GraphPad Prism version 8.0.1 for windows (GraphPad Software Inc, San Diego, CA). Data is presented as means ± standard deviation (SD), unless otherwise mentioned. Statistical significance between two groups was determined by using an unpaired *t* test. Statistical significance between two independent groups with more than one dependent variable was compared using multiple *t* tests. Pearson chi-square tests for independence (categorical variables) were used to examine differences in ulcer status and diarrheal status between treatment groups. Logrank test was used for comparative analysis of survival rates. Differences were considered to be statistically significant if *p* < 0.05.

## Data Availability

Complete dataset for this study will be made available upon reasonable request to the corresponding authors.
